# Supplementary material for: Relationships between structure, in vivo function and long-range axonal target of cortical pyramidal tract neurons
Source: Nat Commun. 2017 Oct 11;8:870. doi: 10.1038/s41467-017-00971-0 (PMC5636900; doi:10.1038/s41467-017-00971-0)
Supplement: Supplementary file 1 — Supplementary Information [file 41467_2017_971_MOESM1_ESM.pdf]

Supplementary Figures

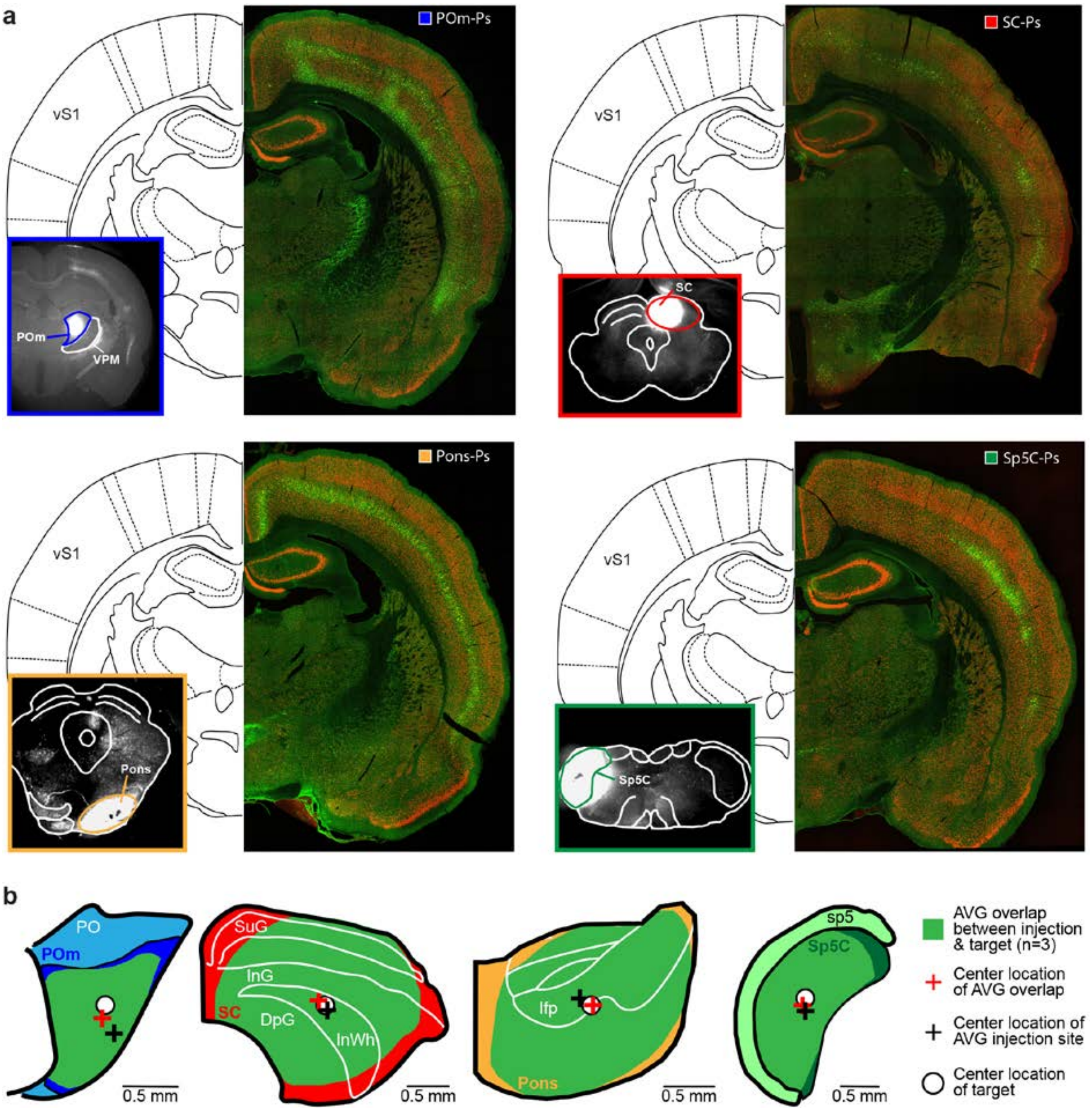

Supplementary Figure 1: a. Exemplary images illustrating the four subcortical injection sites, investigated here, and the respective cortex area- and layer-specific retrograde labeling of PTs. These images correspond to those shown in **Fig. 1b**. **b.** Quantification of the location and overlap of injections sites with the respective target areas (n=3 for each target). To do so, images of the injections sites were aligned with corresponding images from the Paxinos Rat Brain Atlas (see Methods). The outlines of the target areas represent those from the atlas. On average, injections sites overlapped to 90±8% with the target areas and the centers of the overlap area were on average ~100 µm away from the center locations of the respective target area. The ‘average’ injection/target overlaps (green areas) correspond to the average soma distributions shown in **Fig. 1b**.

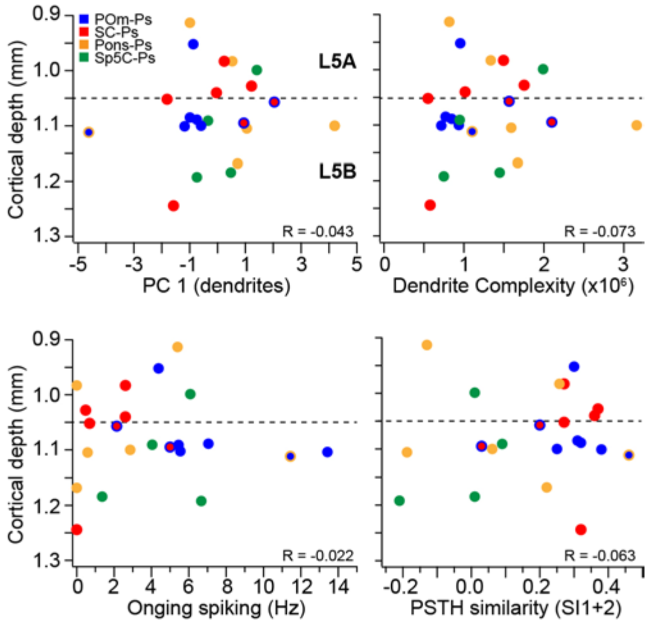

Supplementary Figure 2: Quantification of the target-related structural and functional properties with respect to the PTs' respective soma depth location. None of the parameters correlated with cortical soma depth (i.e. after registration), and PTs were not different depending on their respective soma locations within L5A or L5B.

Supplementary Tables

Dendritic features	POm (n=5)	POm+dual-P (n=6)	Pons (n=5)	Sp5C (n=4)	SC (n=5)	SC+dual-Ps (n=7)	1-way ANOVA
Soma depth (µm)	1065 ±64	1073 ±60	1054 ±103	1116 ±91	1079 ±89	1071 ±83	0.76
**All dendrites**							
Length (µm)	12711 ±1230	13044 ±1369	16785 ±4692	13794 ±3776	14217 ±4264	14666 ±4070	0.32
BB (10^7 $µm^3$)	26.3 ±6.2	25.2 ±6.2	26.3 ±8.9	20.7 ±9.6	23.6 ±11.3	23.8 ±10.4	0.37
BB hori. Extent (µm)	644 ±86	629 ±85	649 ±70	565 ±133	613 ±147	618 ±134	0.35
Branch points	66 ±3	68 ±5	98 ±24	90 ±15	81 ±19	84 ±19	0.05
Avg branch order	6 ±2	7 ±2	8 ±2	8 ±4	6 ±2	6 ±1	0.34
Max branch order	16 ±6	17 ±5	19 ±3	21 ±8	16 ±3	15 ±3	0.31
Avg endp.-soma (µm)	428 ±45	429 ±41	427 ±91	398 ±41	362 ±49	365 ±45	0.28
Max endp.-soma (µm)	1083 ±58	1082 ±52	1059 ±113	1117 ±79	1055 ±51	1048 ±50	0.40
Sum endp.-soma (µm)	31418 ±3414	32080 ±3457	46566 ±19340	39135 ±8838	33014 ±10265	34235 ±9912	0.17
CMS-soma (µm)	281 ±49	284 ±44	279 ±95	283 ±77	222 ±71	228 ±66	0.36
**Apical dendrites**							
Length (µm)	7752 ±739	8084 ±1048	10597 ±3715	8514 ±1415	8578 ±2836	8867 ±2700	0.27
BB (10^7 $µm^3$)	20.7 ±4.3	20.3 ±4.0	22.9 ±7.7	18.0 ±8.5	18.9 ±10.5	19.2 ±9.6	0.25
BB hori. extent (µm)	627 ±86	615 ±82	641 ±85	559 ±136	590 ±151	598 ±140	0.32
BB vert. extent (µm)	1101 ±74	1112 ±71	1082 ±110	1135 ±91	1068 ±38	1059 ±43	0.50
CMS-soma (µm)	506 ±82	502 ±74	496 ±120	494 ±141	401 ±91	413 ±89	0.31
**Basal dendrites**							
Length (µm)	4959 ±860	4960 ±769	6188 ±1507	5280 ±2418	5639 ±2219	5799 ±2069	0.71
BB (10^7 $µm^3$)	5.1 ±1.9	4.9 ±1.8	4.3 ±2.0	3.2 ±1.7	3.9 ±1.9	3.9 ±1.7	0.52
BB hori. extent (µm)	532 ±89	518 ±87	530 ±57	476 ±85	515 ±78	520 ±72	0.69
BB vert. extent (µm)	354 ±48	359 ±44	297 ±82	273 ±68	278 ±56	278 ±51	0.22
Branch points	24 ±5	24 ±4	37 ±6	33 ±14	32 ±11	34 ±11	0.25

Supplementary Table 1. Soma-dendritic features (mean ± SD) that discriminate thick-tufted (PTs) from slender-tufted (ITs) morphologies in L5 of rat vS1. Principal components of these parameters are shown in **Fig. 3b**. For a detailed description of each parameter see[3, 20]. In

brief: (1) pia-soma distance along the vertical cortex axis after registration to the D2 barrel column (soma depth); (2) total dendrite path length (length); (3) volume of the bounding box around all dendrites (BB); (4) lateral extent of the dendrite BB (hori. extent); (5) number of bifurcations (branch points); (6) depth of the dendritic tree averaged across all dendrites (avg branch order); (7) deepest branch order (max branch order); (8) direct Euclidean distance between dendrite ending points and soma, averaged across all ending points (avg endp.-soma); (9) maximal ending point to soma distance (max endp.-soma); (10) sum of all endpoint to soma distances (sum endp.-soma); (11) Euclidean distance between the dendrites center of mass and the soma (CMS-soma); (12) path length of the apical dendrite; (13) volume of the bounding box around the apical dendrite; (14) lateral extent of the apical dendrite BB; (15) vertical extent of the apical dendrite BB (verti. extent); (16) Euclidean distance between the apical dendrite's center of mass and the soma; (17) path length of basal dendrites; (18) volume of the bounding box around all basal dendrites; (19) lateral extent of the basal dendrite BB; (20) vertical extent of the basal dendrite BB; (21) number of bifurcations along basal dendrites. The rightmost column represents P-values from 1-way ANOVA test of the four groups without dual-Ps. The significant difference in the number of branch points originates from the group of POm-Ps that have in general less branch points than PTs projecting to the other targets (see Results).

Target	Pons	Sp5C	POm	SC
**Pons**	**276**	40	82	50
**Sp5C**	35	**220**	4	28
**POm**	55	5	**146**	24
**SC**	55	20	23	**72**

Supplementary Table 2. Numbers of PTs per average C2 barrel column. Analogous to **Fig. 1f-g**.
