# Supplementary material for: Relationships between structure, in vivo function and long-range axonal target of cortical pyramidal tract neurons
Source: Nat Commun. 2017 Oct 11;8:870. doi: 10.1038/s41467-017-00971-0 (PMC5636900; doi:10.1038/s41467-017-00971-0)
Supplement: Supplementary file 3 — Description of Additional Supplementary Information [file 41467_2017_971_MOESM3_ESM.pdf]

Description of Additional Supplementary Files

File Name: Supplementary Data 1
Description: Target prediction tool
