# Supplementary material for: Relationships between structure, in vivo function and long-range axonal target of cortical pyramidal tract neurons
Source: Nat Commun. 2017 Oct 11;8:870. doi: 10.1038/s41467-017-00971-0 (PMC5636900; doi:10.1038/s41467-017-00971-0)
Supplement: Supplementary file 4 — Supplementary Data 1 [file 41467_2017_971_MOESM4_ESM.zip › PT_SubCtx_Target_Predictor_Final/README.docx]

**Tool for predicting the subcortical target of PTs in rat vS1**

This python-based tool allows estimating the most likely subcortical target – POm, SC/Pons or Sp5C – of individual *in vivo* recorded (and reconstructed) PTs in rat vS1, based on measurements of ongoing spike rate, soma depth location and dendrite morphology. The target prediction is possible because neurons that project their long-range axons to these four subcortical target structures represent more than 80% of the PTs in L5 of vS1, and because the three cellular properties result in a structure-function parameter space, where PTs with different targets form largely non-overlapping clusters **(Fig. 4)**. In addition, responses of PTs to air puff stimuli to the whiskers of anesthetized rats can be used to improve this estimate, if experiments are performed under the same condition as described in the present study. The tool has been implemented by Robert Egger. Please contact [Robert.Egger@nyumc.org](mailto:Robert.Egger@nyumc.org) for questions concerning installation and usage of the tool.

**Installation:** Running the tool requires python 2.7, as well as the numpy and scipy libraries to be installed (<https://www.python.org/downloads/>; <http://www.scipy.org/install.html>). Extract all files from the PT_SubCtx_Target_Predictor.zip into a folder. Open the file SubcorticalProjectionProbability.py with a text editor and change line 35 to: prefix = '/path/to/installation/directory/' where /path/to/installation/directory/ should be replaced by the path to the folder where you unpacked these files (note the trailing slash).

**Usage:** Start by double-clicking on the file ***SubcorticalProjectionProbability.py*** (Windows), or by running: ***python SubcorticalProjectionProbability.py*** from the command line (Linux, Windows, OS X).

There are four options how to operate the tool:

1. **Prediction of subcortical target by ongoing spike rate only:** POm-Ps have significantly higher ongoing spike rates than all other PTs in rat vS1. Sp5C-Ps have higher ongoing spike rates than SC-/Pons-Ps (see also **Fig. 3e** and **Table 1**). By providing the tool with the ongoing spike rate of a recorded neuron in L5 of vS1 (in Hz), it is hence possible to obtain a first order estimate of the neuron’s subcortical target.

**Limitations:** Without reconstructing the morphology, it is however in general unclear, whether the recorded neuron belongs to the class of PTs. Even if it is clear that the recorded neuron is located in L5 of vS1, PTs intermingle with inhibitory interneurons and excitatory neurons of other PN cell types. Ongoing spike rates of these other cell types (may) overlap with those of PTs. Further, even though ongoing spike rates of PTs were shown to be not significantly different in anesthetized and awake rats, the present classification is based on recordings in anesthetized animals only. Hence, we cannot exclude that the target-related distributions of ongoing spike rates may be quantitatively and/or even qualitatively different, depending on the experimental condition. Therefore, the first option of the tool should be applied with caution, and can only yield reasonable predictions if the recorded neuron is confirmed to be a PT. In this case – and for anesthetized rats – the accuracy (confidence) of predicting the correct subcortical target is ~77%. The confidence will be provided by the tool as output.

1. **Prediction of subcortical target by ongoing spike rate and soma depth:** Somata of PTs with different subcortical targets form two overlapping sublayers within L5B of rat vS1 (see also **Fig. 1b** and **Table 1**). Soma depth locations and ongoing spike rates correlate with each other, i.e. PTs with ongoing spike rates higher than ~5 Hz that are located within the upper sublayer are typically POm-Ps, and those with lower ongoing spike rates are typically SC-/Pons-Ps. Similarly, Sp5C-Ps can be discriminated from SC-/Pons-Ps in the lower sublayer. Thus, combining the ongoing spike rate of an identified PT in L5 of rat vS1 with precise measurements of its soma depth location will in general improve the confidence at which the neuron’s subcortical target can be predicted. In addition to the ongoing spike rate in Hz, the soma depth location (i.e. vertical distance from the pial surface to the soma) can be provided as a second input parameter in micrometers.

**Limitations:** Same as above. Further, it should be noted that the soma depth location needs to be reconstructed with respect to anatomical landmarks (i.e. pial surface, white matter tract and barrel field in L4), to allow registration to a geometrical reference frame of rat vS1 (registration routines and the reference frame can be obtained from ^1^). Registration allows determining the soma depth location with a precision of ~50 µm. Estimates of the soma depth location by measuring the microdrive depth of the recording pipette (i.e. recording depth) are less precise^1^, ~200 µm. Measuring the recording depth will thus be insufficient to resolve the two target-related sublayers in L5B, whose respective soma density peaks are separated by ~100 µm. Therefore, the second option of the tool can only yield reasonable predictions if the recorded neuron is confirmed to be a PT and if its soma location has been reconstructed and registered to the geometrical reference frame. In this case – and for anesthetized rats – the confidence of predicting the correct subcortical target is ~84%.

1. **Prediction of subcortical target by ongoing spike rate, soma depth and dendrite distribution:** PTs in rat vS1 that project long-range axons to any of the four subcortical target areas investigated in the present study, were shown to have thick-tufted dendritic morphologies, which discriminates PTs from the slender-tufted morphologies of ITs in L5 (see also **Fig. 3b** and **Table 1**). Further, the 3D dendrite distribution across different cortical layers correlated with the subcortical targets areas of PTs. Hence, reconstruction and registration of the 3D dendrite morphology of a recorded neuron allows determining whether the recorded neuron in L5 indeed belongs to the class of PTs (i.e. abolishing the major limitations of the first two options), and additionally provides an improved estimate of the potential subcortical target area when combined with ongoing spike rate and the soma depth location.

**Limitations:** The dendrite morphology (and soma location) needs to be reconstructed and registered with respect to the anatomical landmarks of the geometrical reference frame. The morphological features that are required as input to the tool have been described in detail, previously^2, 3^. An example of the input format for the morphological parameters can be found in the file called: ***example_parameters.csv***. Note: this third option of the tool should be regarded as the default option, as it provides the most reliable target prediction and assures that the classified neuron indeed belongs to the class of PTs. In this case – and for anesthetized rats – the confidence of predicting the correct subcortical target is ~86%.

**4. Prediction of subcortical target by ongoing spike rate, soma depth, dendrite distribution and sensory-evoked response:** In addition to target-specific ongoing activity, soma depth and dendrite distributions, PTs in rat vS1 display sustained activity patterns in response to multi-whisker air puff stimuli that are related to their long-range axonal target **(Fig. 5)**. Hence, recording whisker-evoked spiking responses of PTs as described in the present study in combination with measurements of ongoing spike rate, soma depth and dendrite distribution allows improving the target prediction.

**Limitations:** The sustained activity pattern of PT should be recorded under the same conditions as described in the present study (i.e. air puff of 700 ms duration, 2 s inter-stimulus interval). Note: recordings were performed under urethane anesthesia and it is not known if sensory-evoked responses to air puff stimuli remain unchanged and related to the subcortical target of PTs across different anesthetized/arousal states of the animal. Hence, this option should only be used if the experimental conditions meet those of the present study. In that case, sensory-evoked responses should be provided as PSTHs (in Hz) with 5 ms bin size and including 200 ms of ongoing activity preceding the stimulus, and 600 ms following stimulus offset. An example of the input format for the sensory-evoked responses can be found in the file called: ***example_PSTH.csv***. Based on the present sample, incorporating sensory-evoked responses improved the target prediction to ~95%.
